# *Crocus sativus* Extract as a Biological Agent for Disease-Modifying Therapy of Collagenase-Induced Mouse Model of Osteoarthritis

**DOI:** 10.3390/life13040894

**Published:** 2023-03-28

**Authors:** Blagovesta Boneva, Andrey Marchev, Kristiana Amirova, Petya Ganova, Milen Georgiev, Andrey Tchorbanov, Nikolina Mihaylova

**Affiliations:** 1 Laboratory of Experimental Immunology, Institute of Microbiology, Bulgarian Academy of Sciences, 1113 Sofia, Bulgaria; 2 Laboratory of Metabolomics, The Stephan Angeloff Institute of Microbiology, Bulgarian Academy of Sciences, 139 Ruski Blvd, 4000 Plovdiv, Bulgaria; 3 Laboratory of Immunohistochemistry and Immunopathology, Institute of Microbiology, Bulgarian Academy of Sciences, 1113 Sofia, Bulgaria

**Keywords:** collagenase, experimental osteoarthritis, inflammation, *Crocus sativus* L.

## Abstract

Objectives: Osteoarthritis (OA) is an age-related joint disease that involves the degeneration of cartilage and is the most prevalent form of arthritis, affecting a large part of the population. OA is a multifactorial disorder, and no single etiological mechanism has been found to be common to all forms of the disease. Currently used therapies for control of the disease are mainly nonsteroidal anti-inflammatory drugs (NSAIDs) and corticosteroid medications. The aim of this study was to investigate the extract from *Crocus sativus* as a biological disease-suppressing therapy agent. Methods: Balb/c mice were injected intra-articularly with *Clostridium histolyticum* type IA for induction of osteoarthritis. The mice were randomized to five groups: control group, I group (CIOA untreated), II group (CIOA + 100 mg/kg/daily saffron), III group (CIOA + 50 mg/kg/daily saffron), IV group (CIOA + 25 mg/kg/daily saffron). Flow-cytometry analysis was used to study the splenocytes’ phenotype isolated from the treated animals. The serum levels of inflammatory and anti-inflammatory cytokines were analyzed with ELISA. The histological assessment was used to analyze the saffron extract effect on histopathological alterations. Results: Saffron treatment significantly decreased osteoarthritis-associated joint histological manifestations and decreased serum TNFα levels. The flow-cytometry analysis showed a decrease in pro-inflammatory immune cell subtypes in the spleen. Conclusions: The results obtained suggest that saffron affected the disease progression and could be a potential therapeutic approach in osteoarthritic patients’ therapy.

## 1. Introduction

Osteoarthritis (OA) is a painful disease and one of the most common chronic disabilities in adults. Global trends showed a significant increase in the cases of OA—about 114.5% from 1990 to 2019. The incidences increased with age and showed a female predominance [1]. This slowly progressing degenerating disorder influences the quality of life of affected individuals and leads to enormous costs in the healthcare system [2]. OA severity varies from localized to chronic inflammation based on factors such as age, genetic predisposition, obesity, trauma, congenital, and skeletal deformations of the knee [3,4,5,6,7]. It is characterized by erosion of articular cartilage, proliferation of normally non-dividing chondrocytes, formation of osteophytes, sclerosis of subchondral bone, and synovial inflammation (synovitis) [8,9]. The onset of OA disease is thought to be due to an imbalance between the cartilage degradation and repair process. Synovitis correlates with the pathogenesis and progression of OA [10], and the inflammatory environment is the key factor for the initiation and aggregation of cartilage lesions.

The necessity of the study of the pathological features of OA and the development of new therapeutic strategies leads to the development of mouse models with a pathology comparable to humans and ability to reproduce OA symptoms. Mouse models used in OA research can be categorized into primary/spontaneous [11,12,13] or secondary/induced models [12,14,15]. Van der Kraan et al. developed a model of “secondary” OA using an intra-articular injection with collagenase into the knee joints of mice [16]. This arthritis model has been characterized by severe degenerative cartilage lesions, sclerosis of subchondral bone, osteophyte formation, and consequent deformation of knee joints. The injected collagenase damaged the collagen type I-containing joint structures such as tendons, ligaments, and menisci. All these processes lead to an unstable knee joint and result in osteoarthritic joint lesions [17,18].

Current therapeutic strategies seek to achieve symptom control, prevent and ameliorate pain, and increase mobility. For years, the main approach for the management of OA has been administration of nonsteroidal anti-inflammatory drugs (NSAIDs) medication and corticosteroid injections [19,20]. Some recent studies suggest as therapeutic agents disease-modifying osteoarthritis drugs (DMOAD) [21,22] and some targeting therapies against inflammatory mediators such as IL1, IL6, or TNFα [23,24,25]; however, they are not officially recognized.

To date, there is no effective drug that is able to inhibit damage to the articular structure, reduce long-term disability, or relieve pain. There is an urgent need for the development of a new, noninvasive treatment of OA with an acceptable benefit-to-risk profile. Recently, researchers have been looking for alternatives for OA treatment that possess the advantages of being both natural and safe. Herbal preparations are the most commonly used kind of therapy as alternative medicines for RA. Numerous studies have focused on the therapeutic roles of natural products by isolating and investigating the bioactive components from natural sources such as vegetables, fruits, and herbs. Some natural compounds have properties of enzyme inhibitors and antioxidants and have been reported to have anti-inflammatory properties [26,27].

*Crocus sativus* L. (*saffron*) from the *Crocus* genus, which is part of the *Iridaceae* family, is well-known in herbal medicine for its medical properties. It is a flowering plant with a broad spectrum of applications as a coloring agent and is cultivated mainly in Greece, Iran, Morocco, Spain, and India. The dried stigma, called saffron, and its components (safranal, crocin, and crocetin) show anti-inflammatory, antioxidant, analgesic, antihypertensive, anticancer, hypolipidemic, antitussive, anticonvulsant, anxiolytic, antidepressant, and antinociceptive characteristics [28,29,30,31,32,33,34] The study of Rathore et al. has shown that *Crocus sativus* extract would reduce the levels of the pro-inflammatory cytokines, such as TNFα and IL1β, and influence the attenuation of antioxidant enzymes, such as glutathione reductase and superoxide dismutase in adjuvant-induced arthritic mice [35]. Li et al. and other authors have found that crocin administration relieved paw swelling of rats with RA and reduced arthritis score in groups treated with crocin compared to the RA control group. Moreover, crocin treatment significantly decreased the inducible nitric oxide synthase (iNOS) production and the serum levels of TNFα, IL1β and IL6 in RA rats. In addition, it was reported that the elevated levels of the serum inflammatory mediators, such as enzymatic (MMP-3, MMP-13, MMP-9, and HAases), and non-enzymatic factors, (cyclooxygenase-2 (COX-2), nuclear transcription factor κB (NF-κB), PGE2, and ROS), were highly reduced [36,37,38].

The aim of this study is to investigate the anti-inflammatory and anti-arthritic effects of the crude extract of *Crocus sativus* L. (*saffron*) in the collagenase-induced mouse model of osteoarthritis (CIOA). Our hypothesis is that the saffron extract possesses the potential to have a beneficial effect on the disease course and to suppress the inflammation in the joints.

## 2. Materials and Methods

### 2.1. Mice

Groups of 8–10 weeks old female Balb/c mice (obtained from The Jackson Laboratory, Bar Harbor, ME, USA 04609) were housed in a barrier-type animal facility under specific-pathogen-free (SPF) conditions receiving food and water ad libitum and maintained on 12 h light cycles. All animal procedures and study protocols were approved by the Animal Care Commission at the Bulgarian Food Safety Agency (BFSA) (Licence #306) and were conducted in accordance with the International regulations (EU Directive 2010/63/EU) for the Care and Use of Laboratory Animals.

### 2.2. Crocus Sativus Extract Preparation

Saffron stigmas (*Crocus sativus* L.) grown in the village of Bilgya of Absheron peninsula, Azerbaijan, were used for the preparation of the extract. An alcoholic extract of saffron stigma (kindly provided by Prof. Ulduz Hashimova, Garayev Institute of Physiology of Azerbaijan National Academy of Sciences, Azerbaijan) was prepared as follows: 5 g of dry shredded stigmas of saffron were subjected to alcohol extraction with 75% ethyl alcohol and aged for 2 days in the cold, constantly mixing with a magnetic stirrer. The alcohol solution was filtered, the residue was washed with 75% alcohol and filtered again, then the alcohol was distilled. The resulting liquid extracted under vacuum was concentrated to a dry residue at a temperature of 40–50 °C. The yield of the active compound in the extract relative to the feedstock was about 56% of a viscous resinous substance. Part of the lyophilized extract was stored at −20 °C until the NMR and HPLC analyses were performed. The final solution for animal treatment based on saffron stigmas extract was obtained by its dissolving in phosphate-buffered saline, pH 7.4 (PBS).

### 2.3. Metabolite Profiling through Nuclear Magnetic Resonance (NMR) and High-Performance Liquid Chromatography (HPLC) Analyses of the Crocus Sativus Extract

#### 2.3.1. Analytical Standards and Solvents

Analytical standards of crocin 1 (#80391; crocetin bis (gentiobiosyl) ester, purity ≥ 95%), crocin 2 (#80392; crocetin gentiobiosylglucosyl ester, purity ≥ 95%), and picrocrocin (#89256; purity ≥ 95%) were purchased from PhytoLab GmbH & Co. KG (Vestenbergsgreuth, Germany), while safranal (2,3-dihydro-2,2,6-trimethylbenzaldehyde, purity ≥ 90%) was purchased from Merck KGaA (Darmstadt, Germany). Solvents, such as HPLC grade acetonitrile, methanol, and formic acid were supplied by Merck KGaA.

#### 2.3.2. Metabolite Profiling through NMR

Metabolite profiling was performed according to the protocol described by Koycheva et al. [39]. The extract (10 mg) was dissolved in 0.4 mL CD_3_OD and 0.4 mL D_2_O, buffered to pH 6.0 with KH_2_PO_4_, containing TSPA-d4 (0.005% (*w/v*) final concentration) as internal standard. The ^1^H NMR and 2D NMR spectra (J-resolved, HSQC and COSY) were recorded at 25 °C on an AVII+ 600 spectrometer (Bruker, Karlsruhe, Germany) operating at a frequency of 600.01 MHz with relaxation time 4.07 s and CD_3_OD as an internal lock. The spectra were automatically reduced to ASCII files using AMIX software (version 3.7, Bruker) and phase- and baseline-corrected and referenced at 0.0 ppm to TSPA-d4 using MestReNova software (version 12.0.0, Mestrelab Research, Santiago de Compostela, Spain). All signals were normalized in relation to the peak of TSPA-d4 and scaled to 1.0.

#### 2.3.3. HPLC UV-VIS Analysis

Quantitative analysis of the major identified secondary metabolites through NMR was performed using a Waters HPLC system as previously described [39]. All reference standards were dissolved in methanol, while the saffron extract was prepared in 5 mg/mL solution, dissolved in 75% aqueous methanol. The quantitative analysis of picrocrocin, crocin 1, and crocin 2 were performed according to Valle García-Rodríguez et al. with several modifications [40]. The mobile phases were 0.3% aqueous formic acid (phase A) and methanol (phase B) using a flow rate of 0.8 mL/min with the gradient as follows: phase A was 80% from 0 to 5 min, decreased from 80 to 40 (5–15 min), held 40% from 15 to 20 min, increased to 80% from 20 to 25 min, and held 80% from 25 to 30 min. The detection of crocin 1 and crocin 2 was performed at λ_1_ of 440 nm, while that of picrocrocin was at λ_2_ of 250 nm. The quantitative analysis of safranal was performed according to Kabiri et al. with the following modifications [41]. The mobile phases used were deionized water (phase A) and acetonitrile (phase B) using a flow rate of 1.0 mL/min with the following gradient: phase A was 80% from 0 to 5 min, decreased from 80 to 20 (5–25 min), and increased to 80% from 20 to 30 min. The detection wavelength was 303 nm.

The HPLC methods were validated in terms of linearity and sensitivity, based on limit of detection (LOD) and limit of quantification (LOQ). An external standard calibration methodology was applied. All reference standards were dissolved in methanol, and standard solutions from 10–200 µg/mL for picrocrocin, crocin 1, and crocin 2 and from 10–100 µg/mL for safranal were prepared and filtered through 0.45 µm syringe filters for standard curve preparation. The analyses were performed in triplicates (for each concentration), and the calibration curve was constructed as a relationship between the peak areas and the used concentrations of the standard. The linearity of the curve was estimated according to the obtained regression equations *y = ax + b*, where *y* and *x* are the peak area and the concentration of the compound and its relevant coefficient of determinations (*R*^2^). The sensitivity was determined considering that LOD = 3.3 *σ/S* and LOQ = 10 *σ/S*, where *σ* is standard deviation of the response and *S* is the slope of the calibration curve.

### 2.4. Antibodies

Anti-mouse fluorescein isothiocyanate (FITC)-conjugated CD8 (clone 53–5.7), Ly6G (clone 1A8), CD25 (clone PC61), and CD335 (clone 29A1.4); anti-mouse Pacific Blue—conjugated CD3 (clone 145-2C11); anti-mouse phycoerythrin (PE)—conjugated F4/80 (clone 145-BM8), CD69 (clone H1.2F3), CD19 (clone 6D5), and CD 107a (LAMP-1) (clone 1D4B); anti-mouse allophycocyanin (APC)—conjugated CD4 (clone GK1.5), and CD11b (clone M1/70) (BioLegend, Amsterdam, The Netherlands) were used for fluorescence-activated cell sorting (FACS) experiments.

### 2.5. Induction of OA

The induction of OA was performed as described [42]. Briefly, after anesthesia the animals were injected intra-articularly (i.a.) with 10 µL/per knee (2 mg/mL) collagenase solution from Clostridium histolyticum type IA in TRIS-HCl, pH 7.0 (Sigma-Aldrich, Darmstadt, Germany, #C9891). A control group of animals was injected intra-articularly with 10 μL PBS per knee.

### 2.6. Treatment Schedule

One day after the collagenase injection, the animals were randomized into four groups (five mice per group). The control group of collagenase-injected animals (I group) received per os 300 µL PBS/mouse daily. Three other groups were treated per os with different concentrations of saffron extract for 30 days: II group—treated with 100 mg/kg/daily; III group—treated with 50 mg/kg/daily; IV group—treated with 25 mg/kg/daily (300 µL per mouse). The healthy Balb/c mice were treated every day with 300 µL PBS/mouse.

### 2.7. Isolation of Splenocytes

The spleens from the experimental animals were taken thirty days after the collagenase challenge, and single-cell suspensions were prepared by grinding through sterile cell strainers (BD Biosciences, Erenbodegem, Belgium). Erythrocytes were lysed with hypotonic lysis buffer (150 mM NH_4_Cl, 10 mM KHCO_3_, 0.1 mM Na_2_EDTA, pH 7.2), and after two washes with FACS buffer containing 2.5% fetal calf serum (FCS) and 0.05% sodium azide, the lymphocytes were counted and analyzed by flow cytometry.

### 2.8. Flow-Cytometry Analysis

The isolated spleen cells (2 × 10^5^ cells/tube) were washed with FACS buffer and incubated with one of the next anti-mouse antibodies mixes: for B cell gating, CD19 antibody; for gating T cells, CD3, CD4, CD8 antibody mixture was used; for activated effector T cells, CD3, CD4, CD25, and CD69 antibody mixture; for activated cytotoxic T cells, CD3, CD8, and CD107a antibodies; for NK cells, CD3 and CD335 antibodies; for activated NK cells, CD3, CD335, and CD107a mix of antibodies; for macrophages, F4/80 and CD11b antibodies, and for the gating of neutrophils, CD11b and Ly6G antibody mixture was used. The incubation with the antibody mixtures was performed for 20 min on ice. Thirty thousand cells were collected and analyzed from each sample with a BD LSR II flow cytometer using the FlowJo^TM^ v10.1.8 software (Becton, Dickinson & Company, San Jose, CA, USA).

### 2.9. MTT Proliferation Assay

Isolated spleen cells from animals with OA and healthy controls were incubated with different concentrations of the tested saffron extract (ranging from 0.25 to 1.0 mg/mL) in 96-well culture plates for 3 days at 37 °C/5% CO_2_. Cells cultured in medium only were used as controls. For the last four hours of incubation, MTT (3-[4,5-dimethylthiazol-2-yl]-2,5-diphenyltetrazolium bromide; thiazolyl blue) was added (5 mg/mL), the medium was aspirated, and 200 µL dimethyl sulfoxide/per well was used to dissolve the formed formazan crystals. The cell proliferation was assessed by measuring the absorbance at 590 nm with background subtraction at 620 nm.

### 2.10. Cytokine Detection

Sera from all terminal animals were isolated and kept frozen at −70 °C for further analyses. TNFα, IL6, and IL4 levels were measured in mouse sera using ELISA sets (BioLegend, Amsterdam, The Netherlands) and according to the manufacturer’s instructions.

### 2.11. Histological Analysis and Scoring

Groups of mice injected with collagenase IA type and treated with different concentrations of saffron extract as well as healthy controls were sacrificed by cervical dislocation 31 days after intra-articular collagenase injection. The isolated knee joints were fixed in 4% phosphate-buffered formalin (pH 7.4), and after subsequent decalcification in 20% EDTA for 10 days, the specimens were washed, embedded in paraffin (Paraplast Plus^®^, Sigma-Aldrich), and then sectioned. The sections (5–7 mm) were deparaffinized in xylene substitute (Tissue-Tek^®^ Xylene, Sakura Finetek, CA, USA) followed by dehydration in a graded series of ethanol to water.

For general histopathological examination of cell and tissue morphology, hematoxylin and eosin (H&E) staining was performed. For the analysis of the articular cartilage and evaluation of the presence of proteoglycans and glycosaminoglycans, staining with Toluidine blue was performed.

To score the degree of histopathology alterations in the knee joint of the experimental groups, the OARSI Osteoarthritis Cartilage Histopathology Assessment System (scores between 0 and 4) based on histologic features of OA progression was used [43]. Fissuring/lesions: 0—Lack of fissuring on the cartilage surface; 1—Cracking on the cartilage restricted to surface and superficial zone; 2—Lesions extending into the middle zone; 3—Fissuring that stretches to the level of the deep zone; 4—Lesions extending to the deep zone; Chondrone formation: 0—No cluster formation; 1—Two chondrocytes (doublets) within the same lacunae along the surface of the cartilage; 2—Doublets and triplets of chondrocytes within the same lacunae along the cartilage’ surface; 3—3-4 chondrocytes within the same lacunae along the surface site of the articular cartilage; 4—More than four chondrocytes within same lacunae; Vascularity: 0—Normal; 1—Weak increase in the blood vessels in locations over the cut; 2—Mild increase in the number and dilatation of the blood vessels throughout the section; 3—A moderate increase (up to 50%) in the number and dilatation of the blood vessels in the section; 4—A marked increase (more than 50% of the section) in number and dilatation of the blood vessels; Fibrosis: 0—Normal; 1—Weak fibrosis within the section; 2—25% of the section affected of fibrosis; 3—25 to 50% of the section affected of fibrosis; 4—More than 50% of the section affected of fibrosis.

### 2.12. Statistical Analysis

The statistical analysis used was performed with Prism software from GraphPad (San Diego, CA, USA).

For the two-group comparison, for a comparison of more than two groups, and for multivariable analyses, the two-tailed *t*-test or Mann–Whitney test, one-way ANOVA with Tukey post-test or Kruskal–Wallis test, and two-way ANOVA with Bonferroni posttest were used. Values in the figures correspond to mean ± SD. *p* < 0.05 was considered statistically significant.

## 3. Results

### 3.1. NMR and HPLC Analysis of the C. sativus Extract

**Figure 1:**
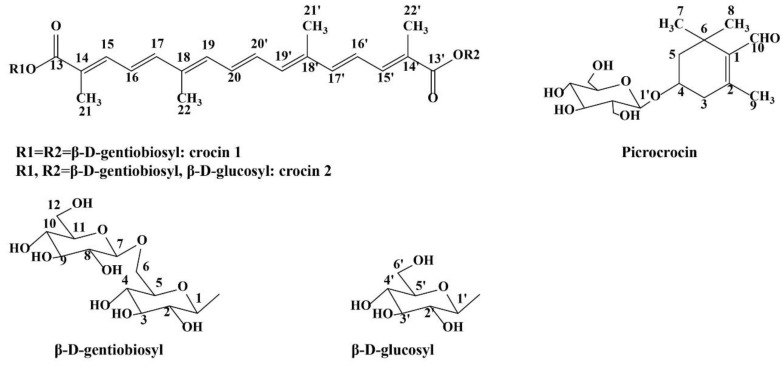
Chemical structures of crocin 1, crocin 2, and picrocrocin.

The characteristic features (color, aroma, and flavor) of saffron are formed by: crocins (glycosylated apocarotenoids, containing glucose, gentiobiose, neapolitanose, or triglucoses saccharidic moieties) responsible for the color; picrocrocin (the glucosylated monoterpene precursor of safranal) responsible for the bitter taste; and safranal (a monoterpene aldehyde derived from the chemical or enzymatic dehydration of picrocrocin during saffron handling, drying, and storage) giving rise to its typical odor and aroma [44]. These structures are presented in Figure 1.

NMR metabolite profiling has received general acknowledgement as a powerful method for quality assessment for a wide range of foods. The NMR spectrum is a type of a fingerprint for a product that has qualitative and quantitative information on its chemical composition. Concerning saffron, NMR investigations have been applied for structural characterizations of crocetin or crocetin derivatives [44]. Here, we used 1D (^1^H NMR) and 2D NMR spectra, such as ^1^H-^13^C heteronuclear single quantum correlation (^1^H-^13^C HSQC), ^1^H-^1^H homonuclear correlation spectroscopy (^1^H-^1^H COSY), and ^1^H-^1^H total correlated spectroscopy (^1^H-^1^H TOCSY), to identify the main secondary metabolites of saffron.

**Figure 2:**
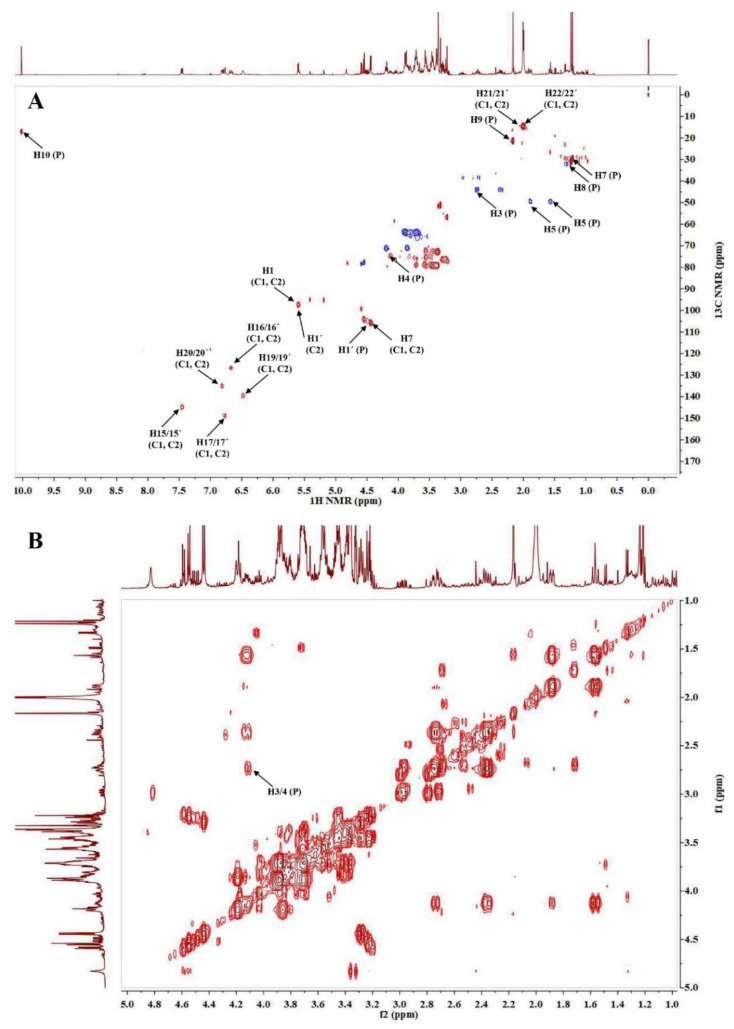
(**A**) ^1^H-^13^C HSQC spectrum of *C. sativus* extract and the characteristic signals of picrocrocin (P), crocin 1 (C1), and crocin 2 (C2); (**B**) ^1^H-^1^H COSY spectrum of *C. sativus* extract and the characteristic signals of picrocrocin (P).

The ^1^H NMR spectrum of saffron in CD_3_OD and D_2_O solution was dominated by the singlet of the aldehydic proton CHO-10 (δ_H_ 10.02, s) and intense singlets from the methyls of picrocrocin at δ_H_ 1.23, δ_H_ 1.24, and δ_H_ 2.15 of 2,6,6-trimethyl-1-cyclohexene-1-carboxaldehyde moiety, as typically observed for picrocrocin. Picrocrocin was also observable through the aldehydic signal and the signals of glucose. According to the ^1^H-^13^C HSQC spectrum, characteristic peaks of δ_H_ 1.23/δ_C_ 26.26 (CH_3_-7; s); δ_H_ 1.24/δ_C_ 28.62 (CH_3_-8; s); δ_H_ 1.57/δ_C_ 46.39 (CH_2_-5; t, *J* = 12.2); δ_H_ 1.89/δ_C_ 46.39 (CH_2_-5; ddd, *J* = 12.7, 3.1, 2.6); δ_H_ 2.15/δ_C_ 19.12 (CH_3_-9; s); δ_H_ 2.74/δ_C_ 41.31 (CH_2_-3; ddd, *J* = 18.9, 5.8, 2.4); δ_H_ 4.12/δ_C_ 71.86 (CH-4, m); and δ_H_ 10.02/δ_C_ 14.20 (CHO-10, s) corresponded to picrocrocin aglycone (Figure 2A). The β-linked glucose was recognized according to the anomeric proton δ_H_ 4.44/δ_C_ 102.79 (H1′, d, *J* = 7.9) [45]. A clear correlation between protons 3 and 4 was observed in the COSY spectrum (Figure 2B) [46].

**Figure 3:**
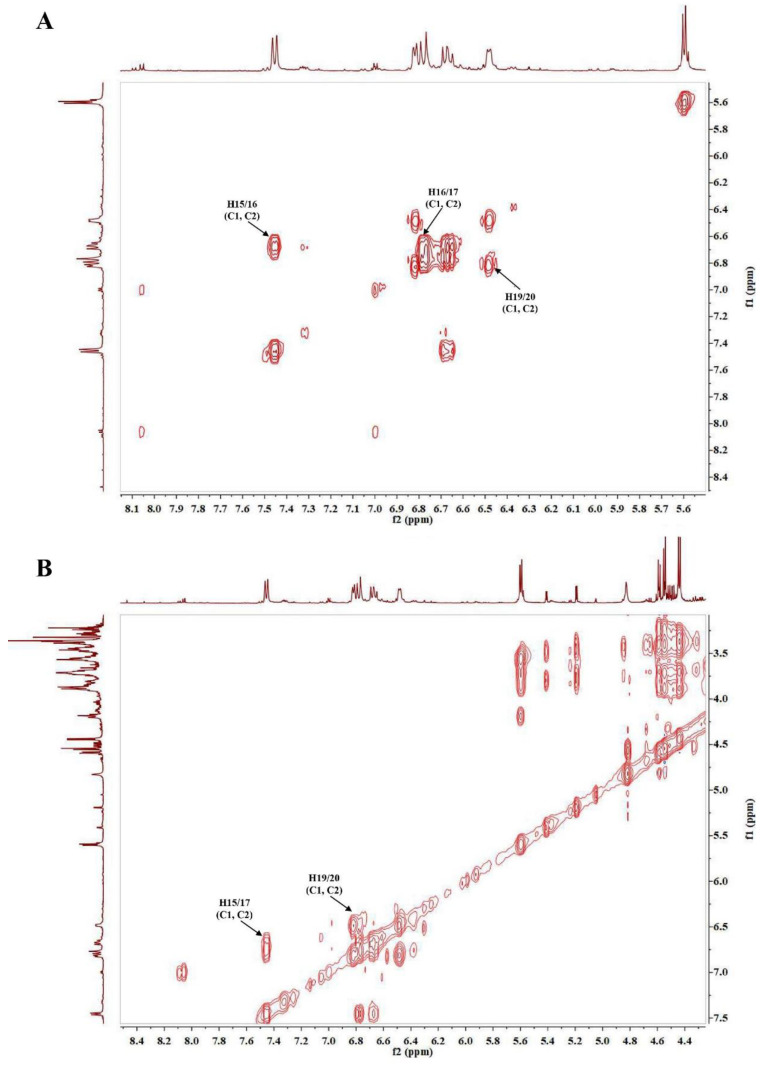
(**A**) ^1^H-^1^H COSY spectrum of *C. sativus* extract and the characteristic signals of crocin 1 and crocin 2 (C1 and C2); (**B**) ^1^H-^1^H TOCSY spectrum of *C. sativus* extract and the characteristic signals of crocin 1 (C1) crocin 2 (C2).

The group of broad signals in the ^1^H NMR spectrum between 6.50 and 7.50 ppm are relative to the conjugated double bonds of crocins. The obtained data confirmed the presence of the dominant content for trans crocins, which was explained by the presence of methyl resonance at 1.99 and 2.00 ppm. Anomeric protons of saccharides bound to crocetin, primarily gentiobiose and glucose, were present in β isomeric form and both occurred at 5.60 (ring A of gentiobiose), 4.44 (ring B of gentiobiose), and 5.62 ppm (for glucose) [47]. Crocin 1 and 2 have a two-fold axis of symmetry (C_2_), making them chiral molecules. The molecule has a carotenoid part with a distinct ABC and AA′BB′ spin systems observed from the coupling patterns, and two types of methyl groups were observed as well. From the HSQC spectrum (Figure 2A), the following carbon to proton correlations were observed: δ_H_ 1.99/δ_C_ 11.64 (CH_3_-21/21′; s); δ_H_ 2.00/δ_C_ 11.05 (CH_3_-22/22′; s), δ_H_ 7.45/δ_C_ 141.86 (CH-15/15′; d, *J* = 11.5); δ_H_ 6.67/δ_C_ 123.63 (CH-16/16′; dd, *J* = 14.5, 15.0); and δ_H_ 6.78/δ_C_ 145.87 (CH-17/17′; d, *J* = 14.7), revealing the ABC spin system. The signals at δ_H_ 6.48/δ_C_ 136.50 (CH-19/19′; m) and δ_H_ 6.82/δ_C_ 132.08 (CH-20/20′; m) were assigned to the AA′BB′ system [47]. In crocin 1 (*trans*-crocetin di (β-D-gentiobiosyl) ester), the sugar gentibiose (two glucose units with a β 1-6 linkage) was identified by the correlations δ_H_ 5.60/δ_C_ 94.71 (CH-1; d, *J* = 7.6) and δ_H_ 4.44/δ_C_ 102.79 (CH-7; d, *J* = 7.9), while in crocin 2 (*trans*-crocetin β-D-gentiobiosyl-β-D-glucosyl ester) the correlations at δ_H_ 5.62/δ_C_ 94.34 (CH-1′; d, *J* = 9.0) revealed the presence the β-linked glucose assigned as R2 [47]. The loss of one glucose residue in one end of the molecule results in the loss of symmetry in the central part of the molecule that contains the conjugated double bonds [48]. The structures of crocin 1 and crocin 2 were also confirmed by their characteristic signals according to the relevant COSY spectrum, revealing the correlations between protons 15/16; 15′/16′; 16/17; 16′/17′; 19/20; 19′/20′ (Figure 3A). A good correlation between 15/17 and 19/20 protons were observed in the TOCSY spectrum (Figure 3B).

The HPLC method used to detect and quantify picrocrocin, crocin 1, and crocin 2 was validated for linearity and sensitivity. The respective calibration curves were as follows: y = 3.13 × 10^4^*x* − 1.47 × 10^4^ (for picrocrocin), y = 1.29 × 10^5^*x* + 1.61 × 10^6^ (for crocin 1), and y = 1.23 × 10^5^*x* + 1.47 × 10^6^ (for crocin 2) had a good linear relationship in the concentration range between 10 and 200 μg/mL and characterized with R^2^ of 0.9963, 0.9547, and 0.9515, respectively. The LOD and LOQ values were 4.22 and 12.78; 4.24 and 12.86; 3.83 and 11.61 µg/mL for picrocrocin, crocin 1, and crocin 2, respectively. The calibration curve for safranal was characterized by an equation y = 8.76 × 10^4^*x* + 4.33 × 10^4^, R^2^ of 0.9879, and LOD and LOQ values of 2.71 and 8.22 µg/mL.

The performed triplicate extraction of 0.9617 g *C. sativus* dry stigmas resulted in 0.5516 g (57.36%) extract yield. According to the HPLC quantification, the content of picrocrocin, crocin 1, and crocin 2 in the *C. sativus* extract resulted in 1.82 ± 0.04; 0.60 ± 0.07; 1.99 ± 0.04 mg/g extract, respectively.

Traditionally, picrocrocin, safranal and crocins, especially trans-crocetin di-(β-D-gentiobiosyl) ester (T-4GG) and trans-crocetin (β-D-gentiobiosyl)-(β-D-glucosyl) ester (T-3Gg), are considered as the respective chemical determinants of the bitterness, scent, and color of saffron and are used to estimate saffron quality [49]. The diversity and ratio of the constituents of saffron depend on many factors, including environmental conditions, drying processes, and storage time. Methods for harvesting and post-harvesting could both have an effect on the metabolic content of saffron [41]. In our case, safranal was not detected in the analyzed extract, or its content was below the limit of quantification. It is a compound formed by enzymatic loss of glucose from picrocrocin and is only formed while saffron is dried. Thus, different drying methods such as shade, sunlight, a traditional heating system, or electric ovens in various regions affect the safranal content [49]. Safranal content has been reported to vary significantly in saffron samples, from amounts under the LOQ to 2.1 mg/g saffron samples originating from different localities in Iran [50]. 

### 3.2. The Impact of Saffron Extract on Cell Populations in Spleens of Mice with CIOA

**Figure 4:**
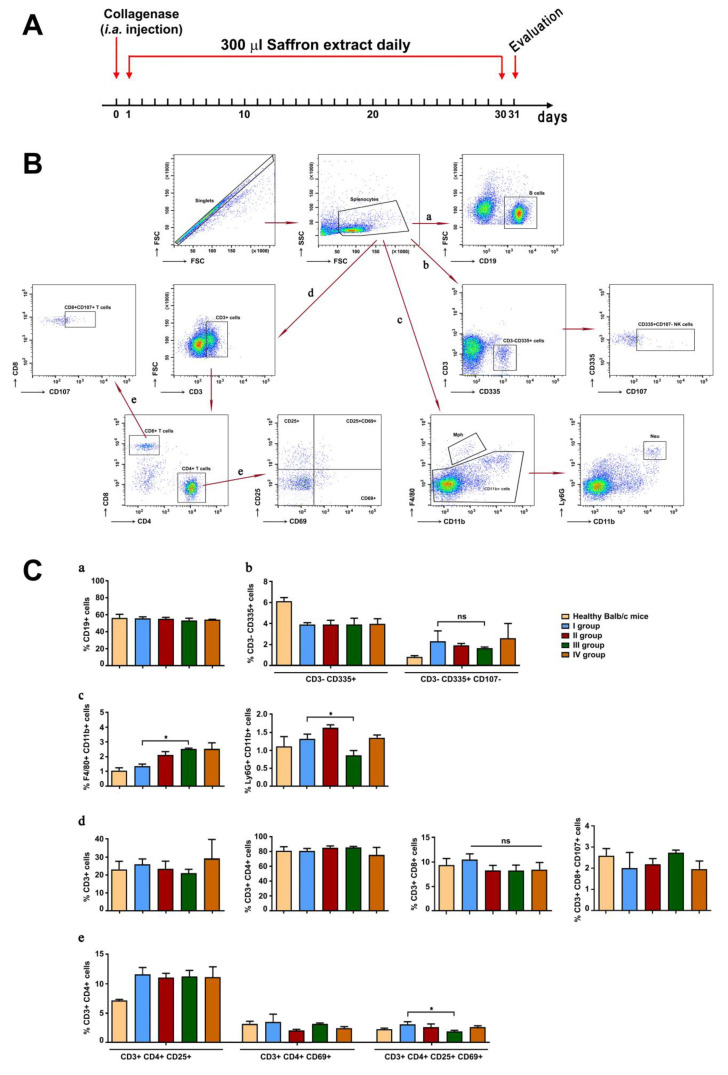
Scheme of treatment (**A**) and gating strategy for flow-cytometry analysis of splenocytes from mice with CIOA, treated with the saffron extract (**B**) Ten thousand cells were analyzed from each sample. Data are representative of at least three experiments. The extracted results from all experiments are presented graphically (**C**). Results are represented as mean ± SD (n = 5). Data were analyzed by the one-way ANOVA test (* *p* < 0.05; ns—not significant).

CIOA was induced in Balb/c mice by i.a. injection of collagenase type IA, and one day later the animals were randomly divided into four groups (see Section 2). The treatment schedule with saffron extract is shown in Figure 4A.

Quantitative evaluation of the main spleen cell populations was performed by FACS analysis. The gating strategy used during the analyses was shown in Figure 4B. The population of CD19-positive splenocytes was not influenced by the treatment with the saffron extract (Figure 4C, a). The activation of the NK cells in collagenase-injected animals (group I) was higher than in the healthy control animals, and the treatment with the different concentrations of the tested extract showed non-significant decrease in CD107 molecule expression (Figure 4C, b). The population of F4/80^high^ CD11b^med^ macrophages have shown a tendency to increase, significant in group III compared to group I (Figure 4C, c, left panel). In contrast, an opposite tendency in the population of Ly6G^high^CD11b^high^ cells was found (Figure 4C, c, right panel).

The quantitative analyses of T lymphocyte populations (CD3 + T cells, CD4 + effector T cells, and CD8 + cytotoxic T cells) did not show a difference after the treatment with the saffron extract, although there was a statistically non-significant tendency to decrease in the CD3^high^ CD8^high^ T cells in all studied groups treated with different extract concentrations (Figure 4C, d, right panel). In group III (mice treated with 50 mg/kg/daily), the effector T cells exhibited decreased expression of the activation markers CD25 and CD69 compared to the untreated collagenase-injected animals (Figure 4C, e, right panel).

### 3.3. Cytokine Measures

**Figure 5:**
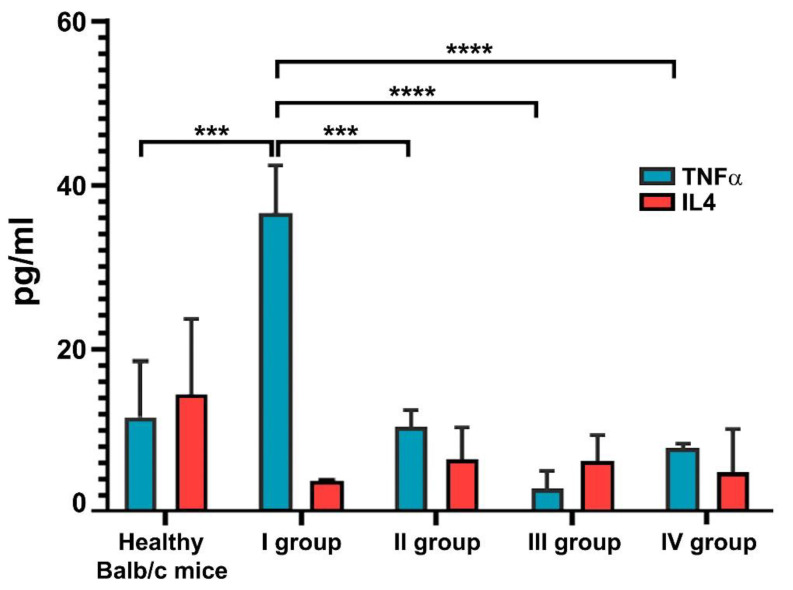
IL4 and TNFα levels in the sera of mice with CIOA and treated with saffron extract. Results are represented as mean ± SD (n = 5). *p* values were calculated using the two-way ANOVA test followed by Bonferroni’s multiple comparison test (*** *p* < 0.001; **** *p* < 0.0001).

The serum levels of IL4, IL6, and TNFα were measured in the sera of Balb/c mice from all experimental groups at the end of the treatment period using quantitative ELISA (Figure 5). The injection of collagenase type IA leads to the significant increase in the TNFα levels compared to the healthy control mice. The treatment with all concentrations of the saffron extract resulted in significantly lower TNFα production compared to the control collagenase-injected group, and this tendency was stronger in the group of mice treated with 50 mg/kg saffron extract.

As a result of the induction of osteoarthritis, the serum levels of IL4 dramatically decreased compared to the healthy mice group. The treatment with the saffron extract shows a slight increase in the IL4 values in the sera of the treated mice, although it was not statistically significant. There were no observed differences in IL6 values between the experimental groups.

### 3.4. Influence of the Extract from C. sativus on Spleen Cell Proliferation

**Figure 6:**
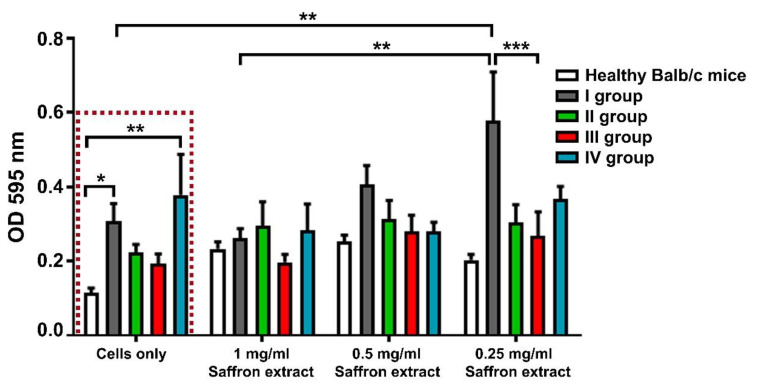
Effect of *C. sativus* extract treatment ex vivo and in vitro on cell proliferation of splenocytes from mice with CIOA detected by MTT assay. Data are represented as mean ± SD; *p* values were calculated using the two-way ANOVA test followed by Bonferroni’s multiple comparison test (* *p* < 0.05, ** *p* < 0.01, *** *p* < 0.001).

The isolated splenocytes were subjected to a cell proliferation test using the studied extract. The spleen cells from CIOA-induced animals had higher metabolic activity compared to cells from healthy animals (Figure 6, red dotted rectangle). We have also observed increased proliferation activity of cells from group I in the presence of in vitro-supplemented saffron extract. There was no difference in the proliferation rates of the experimental groups that were treated in vivo with saffron extract (Figure 6).

### 3.5. Monitoring the Effect of C. sativus Extract on the Pathohistological Changes of the Knee Joint as a Result of CIOA Induction

The ability to suppress or control the pathological changes in the joints during OA development is a very important characteristic for the effectiveness of new compounds for disease treatment. To test the effect of *C. sativus* extract on pathological changes in mice with CIOA, the joints of experimental animals were subjected to histological analysis.

**Figure 7:**
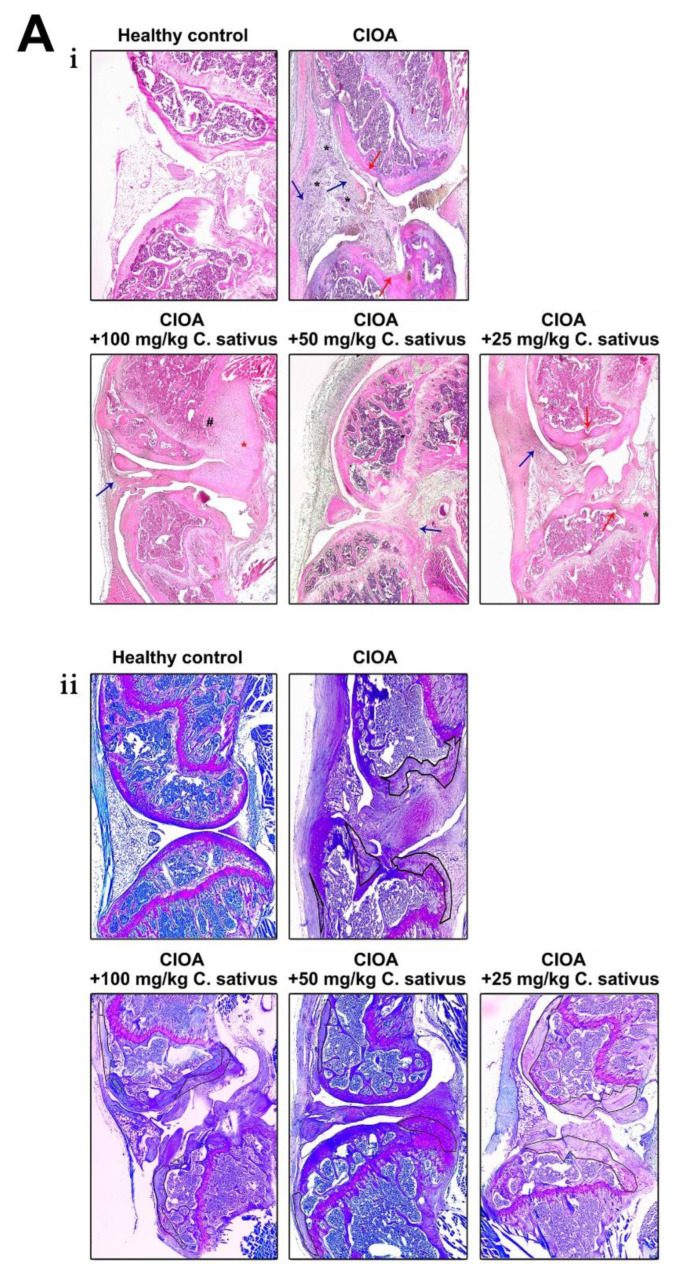

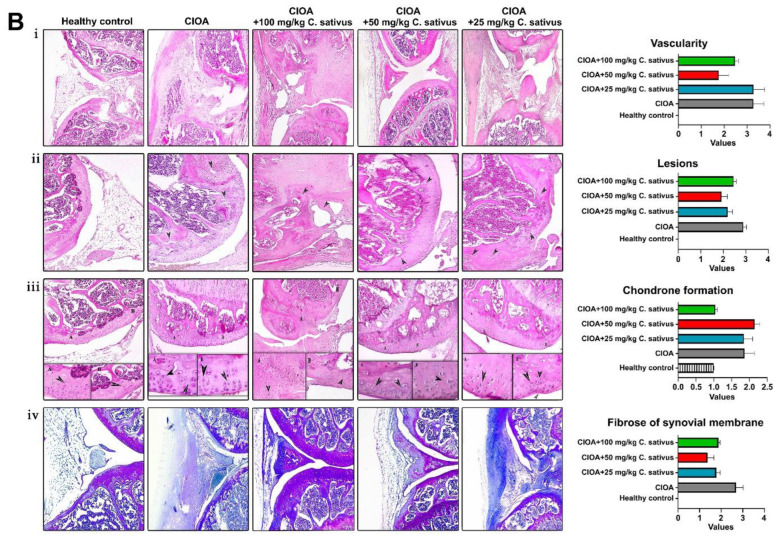
(**A**) Representative histological sections from mice with CIOA after 30 days of treatment with extract from *C. sativus*. i. Hematoxilin–eosin staining; hyperplasia and hypertrophy of the synovial membrane—blue arrow; enhanced angiogenesis—black asterisk; destruction of the cartilage—red arrow; local erosion—#; large osteophyte—red asterisk; ii. Toluidine blue staining; areas of glycosaminoglycan loss—marked areas; (**B**) Representative histological sections and bar graphs of the main histopahtologycal scores from mice with CIOA after 30 days of treatment with extract from *C. sativus* (n = 5); Higher magnifications of the images (20×) shown in (iii) stained with H&E. i. Representative pictures from the groups and vascularity score; ii. Representative pictures from the groups and lesions score; iii. Representative pictures from the groups and chondrone formation score; iv. Representative pictures from the groups and fibrose of synovial membrane score. The microscopic grading system is described under Histological Analysis and Scoring in Section 2. Magnification–5×.

In group I, CIOA-induced animals, the structural changes of the joint presented the main well-defined pathologic signs of osteoarthritis (Figure 7A). Hyperplasia of a large part of the synovial membrane and invasion into the bone and cartilage is also observed. A significant increase in fibroblastic synoviocytes was found, leading to tissue proliferation, especially in areas located near cartilage and bone (blue arrow, Figure 7A, i; Figure 7B, iv). Enhanced angiogenesis is observed, with newly formed blood vessels having an irregular shape and different wall thickness (black asterisk, Figure 7A, i; Figure 7B, i). At the same time, there is an accumulation of a significant amount of fibrous tissue, an indicator of the processes of destruction of the normal, loose connective tissue forming the synovium. Visible destruction of the cartilage is observed (red arrow, Figure 7A, i). It leads to the exposure of the subchondral bone, resulting in contraction with proinflammatory factors produced by surrounding cells, thus causing local bone erosion in this area. Local and generalized bone erosion was presented in the subchondral bone as well as in the cortical bone (arrow, Figure 7B, ii). Clearly distinguishable is destruction in cartilage tissue and loss of glycosaminoglycans (marked areas, Figure 7A, ii). The osteophytic formations in the joint also stand out in a darker blue color.

Histological analysis of animals in group II (treated with saffron extract at a concentration of 100 mg/kg/day) showed well-preserved subchondral bone, with no destroyed areas. In the cortical bone, destruction was observed only in the vicinity of the osteophyte, and generalized bone destruction was suppressed. The local erosion was only found in certain areas (#, Figure 7A, i), and the cartilage is mostly preserved. Bruises and a change in shape were visible, but compared to the animals of group I, it was much more preserved (Figure 7A, i). Hyperplasia and hypertrophy of the synovial membrane and invasion into cortical bone was also noted (blue arrow, Figure 7A, i; Figure 7B, iv). As a result of the inflammatory process, proliferated fibroblastic synoviocytes that fuse with the bone and cartilage were observed. In this group there were large formations such as the one marked with a red asterisk in the figure, which cover the entire joint and make movement difficult. The origin of the large osteophyte cannot be determined, but from its location in relation to the structure of the joint, it most probably belongs to the subtype formed by the cortical bone and fusion with the nearby pannus, enclosing the intra-articular space (red asterisk, Figure 7A, i). There was a loss of GAG in a section of the cartilage of the bones (marked areas, Figure 7A, ii). Despite the loss of glycosaminoglycans, cartilage tissue was much better preserved compared to untreated controls.

In group III (50 mg/kg/day), the synovial membrane was almost in a normal condition (blue arrow, Figure 7A, i). The cartilaginous tissue was well-preserved without visible areas of destruction. Despite the formation of pannus, the form and strength of the cartilage was also well-preserved. There was no destruction, locally eroded areas, or apoptotic chondrocytes or apparent loss of GAG or proteoglycans, which was characteristic of the control group of animals. Generalized bone destruction was suppressed, and subchondral bone was well-preserved together with the cortical bone. The local bone destruction was significantly reduced, and fully preserved cartilage structure was observed (Figure 7A, i). Very small, single areas of glycosaminoglycan loss were found. The presence of osteophytes formed was noted, but the overall shape of the articular cartilage and the overall joint structure were very well-preserved compared to the control CIOA mice (marked areas, Figure 7A, ii).

In the histopathological analysis of animals from group IV (25 mg/kg/day saffron extract), changes in the synovium structure and other major pathological features resulting from the disease were observed (Figure 7A, i). An osteophyte was observed (marked with green asterisks, Figure 7A, i), and compared to the animals treated with 50 mg/kg, the synovium was hyperplastic (blue arrow, Figure 7A, i). Generalized bone erosion was suppressed similar to the higher doses, but in contrast, in the animals of this group, areas of localized bone erosion were found in the subchondral bone (red arrow, Figure 7A, i). There was a loss of GAG in a section of the bone cartilage (marked areas, Figure 7A, ii).

## 4. Discussion

OA is one of the most common joint diseases worldwide, often accompanied with pain and limited movement [51,52]. Its severity varies from localized to chronic inflammation and leads to joint cartilage degeneration, synovitis, and even bone remodeling [53].

At present, there is no effective therapy capable of restoring the damaged structure and function of the cartilage and the synovial tissues in OA. The final stage of treatment for OA is replacement of the joint. Apart from analgesics and nonsteroidal anti-inflammatory drugs (NSAIDs), limitations to conventional medicine to manage OA indicate an increased need for new, safe, and effective therapies for patients with OA. The use of complementary and alternative medicine, including herbal therapies, has the potential to provide a solution to this problem.

In recent years, the efforts of scientists have been focused on studying the effects and mechanisms of action of various natural substances from plant or animal origin. There are a number of studies that prove the positive impact of different plant extracts on the prevention and treatment of osteoarthritis [39]. Saffron, obtained from the dried red stigmas of *C. sativus*, is one of the most expensive spices in the world because of the difficult conditions for stigma production. Saffron is broadly used in the food industry as an additive for coloring and flavoring foods. It is also employed as a drug in traditional medicine. In this current study, we used a collagenase-induced mouse model of OA as a base to test the anti-inflammatory and protective properties of the extract from *C. sativus*.

Saffron is a rich source of multiple active ingredients, including carotenoids, flavonoids, terpenoids, amino acids, and alkaloids [54,55]. These active components show various pharmacological effects such as anti-anxiety, anti-inflammatory, anti-oxidant, antiviral, anti-tumor, hypoglycemic, hypolipidemic, and memory-enhancing properties [56,57].

The development of OA involves immune system activation and leads to inflammation, where the monocytes, neutrophils, lymphocytes, and platelets play an important role [42]. Cytokines are considered to be crucial transmitters of signals for the development of OA and immune cells, such as T cells, macrophages, and synovial fibroblasts, while chondrocytes are involved in the secretion of various cytokines that regulate the inflammatory response. Several studies have revealed elevated levels of pro-inflammatory cytokines, such as TNFα, interferon-gamma (IFNγ), IL1β, IL6, and IL17 in OA [58]. Other immunomodulatory cytokines, including IL4, IL10, IL11, and IL13, have been classified as inhibitory or anti-inflammatory since they reduce the production and/or activation of the proinflammatory cytokines in vitro [59]. The chondroprotective effect of IL4 and IL10 has been shown in vivo [60], and a negative correlation between their expression and immunodetectable TNFα has been reported in OA cartilage [61].

There are a number of studies showing the effect of the saffron extract on the levels of pro-inflammatory and anti-inflammatory cytokines. Zeinali et al. reported a decrease in the serum levels of pro-inflammatory enzymes and cytokines such as COX-2, myeloperoxidase (MPO), phospholipase A2, iNOS, proteinoids, TNFα, NF-κB p65, and ILs such as IL1β, IL6, IL12, IL17A, and IFNγ after treatment with saffron [62]. Similarly, we have also shown that the therapy of CIOA mice with saffron extract leads to a statistically significant decrease in the levels of pro-inflammatory cytokine TNFα in a dose-dependent manner, compared to the CIOA control mice. The animal sera were also tested for anti-inflammatory cytokine IL4 evaluation, and a tendency for elevation of the IL4 levels compared to TNFα was found with best significance at the treatment with 50 mg/kg of saffron (Figure 5).

Macrophages as a major component of the mononuclear phagocyte system play a critical role in OA pathogenesis. In response to microenvironmental stimuli based on their activation, macrophages can be classified as follows: classically activated macrophages (pro-inflammatory, M1) and alternatively activated macrophages (anti-inflammatory, M2). M1 macrophages are responsible for the release of molecules crucial for joint inflammation, while M2 macrophages contribute to tissue repair and resolution of inflammation [63,64]. In OA, the balance between M1 and M2 macrophages could be changed, and the degree of the imbalance was associated with severity of OA. It has been shown that both inflammatory and destructive responses are dependent on macrophages by induction of inflammatory mediators, growth factors, and proteinases [65,66]. Their modulation might be sufficient to moderate OA symptoms and prevent disease progression.

It was shown by Singh et al. that saffron significantly decreased the number of pro-inflammatory macrophages, while the level of anti-inflammatory macrophages increased in a mouse model of colitis [67]. In this study, saffron increased the percentage of F4/80—positive macrophages compared to CIOA untreated control mice. More detailed analysis of the type of macrophage formation is needed, but based on our results, we could hypothesize that the saffron extract may promote the M2 polarization. The treatment of CIOA mice with the studied extract also led to the decrease in the CD3 + CD8 + T cell subpopulation and to the reduction in CD3 + CD4 + T cell activation, especially with 50 mg/kg saffron treatment. Considering that M1 macrophages induce Th1 immune response, we could conclude that saffron may influence the proinflammatory cascade, but additional research is still needed on whether the effect is direct or indirect.

We have already shown that the population of splenic NK cells of CIOA animals was significantly smaller than that in the healthy control group, but highly activated [42]. Yamin et al. have shown that in the synovial fluids of patients with rheumatoid arthritis, NK cells are largely present and are considered important players in bone destruction [68]. The applied therapy with the saffron extract did not affect the percentage of the CD3^low^CD335^hi^ NK cells, but we could observe a decrease in the activation state regarding the expression of CD107a. It has been shown in a murine model of early OA that neutrophils are a significant source of cartilage-degrading enzymes [69]. In our study, we report a decrease in the neutrophil population as a result of the treatment with the saffron extract.

Various studies have shown the anti-proliferative and potent cytotoxic effects of saffron and its components crocin and picrocrocin, and for this reason it is mostly used as a testing agent for the prevention and therapy of cancer [54]. A list of in vitro and in vivo studies shows the significant pro-apoptotic effect on different cancer cell lines [70,71] and the inhibition of tumor progression in mice [72,73].

In recent years, an increased interest in investigating the effects of saffron on joint diseases has been observed. Koski et al. have shown that the administration of crocin to human fetal osteoblasts leads to increased cell proliferation [74]. Here, we have shown that splenocytes from mice with CIOA have higher levels of proliferation ex vivo compared to the healthy controls. Although it is not statistically significant, there is a tendency for regulation of this metabolic activity in the animal groups treated with 100 and 50 mg/kg saffron in comparison with the control untreated CIOA mice. The treatment in vitro with additional doses of saffron did not further affect the proliferation of the treated groups, but it has shown stimulation of cell proliferation of the CIOA mice in a concentration-dependent manner. Additional experiments should be carried out to research this effect.

Comparative analysis of the pathohistological changes in the joints of animals from all experimental groups suggested the conclusion that the treatment with *C. sativus* extract had a beneficial effect on the disease course. The treatment diminished bone destruction and influenced the loss of glycosaminoglycans and proteoglycans. Animals from all three extract therapy groups had preserved articular cartilage compared to the control group of mice.

## 5. Conclusions

With regard to the most important characteristic of OA, it can be stated that treatment with *C. sativus* extract significantly suppressed inflammation in the joints, diminished bone erosion, and helped the preservation of the articular cartilage. The potent components of the tested extract affected the destructive action of osteoclasts and reduced the loss of proteoglycans and GAGs in the joint. All histological findings found in the extract treatment groups together with the data obtained from the serum levels of the cytokines, as well as the data from the proliferative test, indicate that the saffron extract has a beneficial and protective effect in mice with experimentally induced OA. The analyzed extract of *C. Sativus* has shown the therapeutic potential of its bioactive constituents for the treatment of OA.

## Data Availability

Data is contained within the article.
